# Investigating the Relationship between Governance and Key Processes of the Iran IoT Innovation System

**DOI:** 10.3390/s22020652

**Published:** 2022-01-14

**Authors:** Hamze Sadeghizadeh, Amir Hossein Davaie Markazi, Saeed Shavvalpour

**Affiliations:** 1 School of Progress Engineering, Iran University of Science and Technology, Tehran 16846, Iran; h_sadeghizadeh@pgre.iust.ac.ir (H.S.); shavvalpour@iust.ac.ir (S.S.); 2 School of Mechanical Engineering, Iran University of Science and Technology, Tehran 16846, Iran

**Keywords:** Internet of Things (IoT), technological innovation systems (TIS), governance functions, key processes of TIS

## Abstract

Despite the emergence of unique opportunities for social-industrial growth and development resulting from the use of the Internet of Things (IoT), lack of a well-posed IoT governance will cause serious threats on personal privacy, public safety, industrial security, and dubious data gathering by unauthorized entities. Furthermore, adopting a systemic governance approach, particularly for the IoT innovation system, requires a precise clarification on the concept and scope of IoT governance. In this study, by employing the Structural Equation Modeling (SEM) approach, the role of governance in the Iran IoT innovation system is investigated. Contacting respondents across the seven industries, including Information and Communication Technology (ICT), Healthcare, Transportation, Oil and Gas, Energy, Agriculture, and Banking over the course of three months, the authors performed statistical analysis on 319 fulfilled questionnaires using SPPS and Smart PLS software. Findings show that all IoT-related TIS processes have been affected by IoT governance functions. The main result of this study is the proposition of particular governance functions, including policy-making, regulation, facilitation, and service provision with more notable impact on the indicators of the key processes in the IoT-based TIS.

## 1. Introduction

More than two decades after being named by Ashton [1], the Internet of Things (IoT) continues to present opportunities and challenges for communities that want to take advantage of it. Being at the heart of the Fourth Industrial Revolution (Industry 4.0) and the emergence of new applications and services such as smart homes, cities, factories, and industries, in which the starting point is smartening the objects through various sensors, represents the countless opportunities that this technological innovation can create [2]. On the other hand, by first transferring data from intelligent objects through local or global networks, critical challenges such as privacy and security are raised [3]. For seizing these opportunities, developing countries are facing critical challenges at different levels.

The pervasive use of IoT in developing countries will lead to a vital dependency on this technology in coming years. According to previous studies, some countries such as China [4,5], India [6], Indonesia [7], etc., have actively contributed to the IoT development concept as an economic growth driver during recent years. The investigation of national activities and actions in these countries also shows that there is not yet an IoT governance plan in order to decline dependency on developed countries and mitigate the risk of privacy and security.

Iran, as a developing country, is in the emerging stage of IoT [8], and a few previous researchers have addressed the IoT development at the national level without having or proposing a systemic approach. For example, Mohammadzadeh et al. have prioritized the IoT development challenges in Iran in five groups, including Technological, Privacy and Security, Business, Legal and Regulatory, and Cultural challenges [9]. Zarei et al., also have investigated and prioritized Iranian industries in terms of economic prosperity, quality of life, and environmental protection indicators for sustainable IoT development in Iran [10]. It seems that developing countries like Iran should have a systemic approach to the IoT technological innovation at the national level.

The systemic approaches to innovation, including National [11], Sectoral [12], Regional [13], and Technological Innovation Systems (TIS) [14], have tried to structure the process of digital innovations at different levels. TIS, as a socio-technical system is the best choice for addressing the IoT development in terms of paying attention to the critical aspects of this technology. However, the scientific literature on TIS, which originated in developed countries and focused primarily on functional analysis for non-digital industries, has often approached the governance issue as an integrated part of its function instead of interpreting it as an affected concept [15]. What has not yet been considered in the theoretical literature of innovation systems is the adaptation and explanation of governance functions concerning the critical processes of a TIS.

The authors believe that lack of a well-posed IoT governance is more tangible for those researchers and policy-makers who are in developing countries. Because they require a scientific and consequently practical approach to develop digital innovations and technologies that have a profound impact on human and industrial societies [16,17,18]. Although the concept of such governance can be tracked in the literature of TISs in recent years [19,20,21], the existence of ambiguities and uncertainties about the social impact of IoT, especially in developing countries, suggests more in-depth work and indigenous studies are required.

Regarding these, the present study is formed by selecting the case of IoT in Iran to answer this vital question: How does IoT governance at a national level and its functions relate with the IoT innovation system in Iran? To answer this question, the authors have used both qualitative and quantitative methods, respectively, in conceptualization of the IoT governance and its relationship with the key processes of the Iran IoT innovation system. Accordingly, the most important achievement and contribution of this study is the clarification of direct relationships between the IoT governance functions and key processes of the IoT-related TIS.

Following the paper, a conceptual model has been developed after reviewing the theoretical foundations of TIS and IoT governance in Section 2. Research design, methodology, and empirical activities for gathering data are described in Section 3. The statistical results of SEM in Section 4 are considered as inputs for the discussion of outputs in Section 5. Finally, policy and research suggestions are highlighted for those policymakers and researchers interested in the non-technical side of IoT.

## 2. Theoretical Background and Research Model

Since the boundaries of the study are limited to IoT governance on the one hand and TIS on the other, the literature review is divided into two subsections; IoT governance functions and key processes for the IoT-related TIS.

### 2.1. IoT Governance Functions

IoT not only includes applications for human community, but also contains a significant range of services interacting with machines, robots, and equipment. The scale of transmitted data also varies from very small sizes to big data [22] that mainly relate to the privacy of community members. Transferring data through local or global internet-based networks may lead to security threats at the individual and social levels. This is one of the critical challenges of applying IoT at each scale [23]. These concerns, along with the infrastructural challenges in developing countries that are highly dependent on leading countries, illustrate the importance of IoT governance and an appropriate approach at the national level [24,25].

In terms of data usage, Peji’c Bach et al., have investigated the overall usage of big data to form various resources, like IoT as a main source of data alongside the social networks and mobile systems. Additionally, they indicated that the usage of data in some EU countries, such as Finland and Ireland, is remarkable, and other countries, including Italy, Croatia, Germany, and Sweden, are following pioneer countries or are being challenged with this issue [26]. Developing countries which are facing data gathered through IoT applications or services, contrary to developed countries, have critical challenges at a macro level. In India, for example, Chatterjee and Kar have proposed the IoT governance with a particular structure and function focused on policy issues [6]. Additionally, in Iran, as is the case of this study, there has not been any systematic view or governance approach in dealing with not only data, but also big data could be gathered by IoT in the future.

IoT governance is a new and complex concept that was introduced less than two decades ago. In his research, Weber explored IoT governance alongside other challenging issues in European Union (EU) countries, including architecture, identification, privacy and security, standards, and ethics [27]. Supported by society, IoT governance is a framework that addresses critical issues of security and privacy, interoperability, and ethics by understanding the expectations of different stakeholders [28].

In a general sense, Scott defined governance as a wide range of capacities and resources to power on a wide range of governmental, non-governmental, and transnational actors [29]. In his study entitled Modern Government, Benz considered governance as the guidance of all interdependent members under legal systems [30]. Regarding these definitions, governance functions/roles are the key concepts to which researchers have contributed theoretically. They referred to the main functions of governance, including policy-making, regulation, facilitation, and service provision that can be performed with the cooperation of a system’s players [31,32,33,34]. The authors’ belief is that the functions of IoT governance can be the result of these four macro-level functions.

The IoT governance collaboratively: decides on the IoT priorities and executive plans through its policy processes; applies social, technological, and economic interventions through its regulation function; provides the infrastructures and platforms to empower the IoT ecosystem and value chain through its facilitating role; and supplies every product and service to which the stakeholders and society need through the provision function. Thus, the primary hypothesis of the study is that:

**Hypothesis** **1a–d** **(H1a–d).** *The IoT governance has a direct and meaningful relationship with the policy-making, regulation, facilitation, and service provision functions.*

### 2.2. Key Processes for the IoT-related TIS

TIS is a socio-technical system with the primary objective of the development, diffusion, and use of technology or a technological field. The structure and functions of a TIS also can be determined within national, regional, or sectoral boundaries. TIS structure consists of knowledge and products, actors, networks, and institutions [35]. However, to understand the system dynamics, researchers have introduced some key processes or functions for TIS that are necessary to achieve its overall function and purpose [36]. Table 1 presents two common categories of TIS processes with significant overlap, as well as features/criteria for each one in the IoT-related TIS.

It seems that IoT governance and its functions can be conducted in collaboration with the key processes of the IoT-related TIS, if a systemic approach is taken by structural components. As a digital technology under the Industry 4.0, IoT has some commonalities with other digital areas such as big data, cloud computing, artificial intelligence, machine learning, blockchain, etc. Accordingly, the seven functions introduced by Bergek et al. [35] including the ‘‘development of positive externalities’’, explain the relationship between IoT governance functions and key processes of the IoT-related TIS.

Bergek et al. stated that the process of “knowledge development and diffusion” is at the heart of a TIS and is recognized as the knowledge base [35]. Knowledge can develop in various forms such as scientific, technological, production, market, logistics, and design, and consequently provides various stakeholders through diffusion mechanisms [38]. This TIS key process for IoT is very essential in terms of the starting point for developing technical and social solutions to address privacy, public safety, and security issues. Regarding these IoT challenges, particularly at the national level, it seems that some cases such as professional training, conducting feasibility studies, developing complementary technologies, technological and research-based collaborations [37] need governance. Thus:

**Hypothesis** **2a–5a** **(H2a–5a).** *IoT governance functions have a direct and meaningful relationship with the key process of “knowledge development and diffusion” in the IoT-related TIS.*

Acquisition of new technologies, services, and applications in an entrepreneurial way is the foundation of the “entrepreneurial experimentation” process of a TIS [21]. IoT entrepreneurs are dealing with cutting-edge technologies across the four common technical layers, including sensors, networks, platforms, and applications [39], that should be considered by governance. They need dedicated testbeds and incubations for their start-up products and innovative solutions before commercialization [9]. At a higher level, launching pilot IoT projects through the Public-Private-Participations (PPP) can also positively affect the firms’ entrance into the IoT market [37]. Regarding these issues, the study assumed that:

**Hypothesis** **2b–5b** **(H2b–5b).** *IoT governance functions have a direct and meaningful relationship with the key process of “entrepreneurial experimentation” in the IoT-related TIS*.

The relevance and desirability of new technological concepts have great importance in enabling other key processes within a TIS [35]. For a developing country with an economically unbalanced distribution and concentration of resources, the “legitimation” process plays a critical role that needs governance [40]. The power of lobbies, increasing rate of interest groups, acceptance across the society members, and the existence of IoT-related institutions can be considered indicators of governing this key process for IoT technological Innovation system [37,41]. Thus, the authors proposed the following hypotheses:

**Hypothesis** **2c–5c** **(H2c–5c).** *IoT governance functions have a direct and meaningful relationship with the key process of “legitimation” in the IoT-related TIS*.

The evolution and growth of a TIS requires the dedication process of different resources, including human, physical, financial, and spiritual resources entitled “resource mobilization” [35]. Each form of capital can mobilize and be equipped in a unique way that needs to be considered by governance. For example, mobilizing human capital through the specific education programs in a technological field or financial resources through providing Research and Development (R&D) budgets, grants, and loans [42]. One of the more critical aspects which requires physical resources of IoT, especially at the national level, is technical infrastructure and platform in terms of covering privacy, security, and safety challenges [43,44]. It seems that the IoT governance needs to obsessively plan and execute this key process of the IoT-related TIS. Thus, the study proposed a group of hypotheses as follows:

**Hypothesis** **2d–5d** **(H2d–5d).** *IoT governance functions have a direct and meaningful relationship with the key process of “resource mobilization” in the IoT-related TIS*.

Although it is difficult to compete with current concepts for new technologies and innovations, the creation of protected space must be taken into account by the “market formation” process of a TIS. As Hakkert et al. argued, formatting niche markets or considering tax incentives in a short time is an alternative that governance needs to consider [35]. For IoT as a national TIS, the process of market formation consists of providing subsidies to service developers, procuring programs for meeting both the supply and demand sides of the market, and regulation, not only for technical issues through standardization but also for implementing policies made by governance [25,37]. These critical points convinced authors to propose the following hypotheses:

**Hypothesis** **2e–5e** **(H2e–5e).** *IoT governance functions have a direct and meaningful relationship with the key process of ‘market formation’ in the IoT-related TIS*.

The limitation of resources in a TIS leads to the selection of the best options through the “influence on the direction of search” process. This TIS process can collaboratively commit to a variety of system’s players: industry, government, and/or market. Bergek et al. believed that sufficient encouragement/pressure is required to incentivize participations from these three players [35]. The transformation of preferences and expectations of society members, if robust and prominent, can affect the priority of R&D activities and technological transformation [36,45]. Accordingly, setting broad goals for IoT development, the design of favorable rules and regulations, publicizing expectations, and providing direction of development must be governed through this key process for the IoT-related TIS [37,46]. Thus, the study proposed a group of hypotheses as below:

**Hypothesis** **2f–5f** **(H2f–5f).** *IoT governance functions have a direct and meaningful relationship with the key process of “influence on the direction of search” in the IoT-related TIS*.

For both stages of a TIS, formation and growth, it is necessary to run the “development of positive externalities” focusing on external economies. These externalities can also diffuse in different types: pooled labor markets, specialized intermediate goods and service providers, information flows, and knowledge spillovers [35]. The process of “development of positive externalities” for the IoT-related TIS, if well defined by governance, can positively affect other digital technologies under the Industry 4.0, such as big data, cloud computing, artificial intelligence, machine learning, blockchain, etc. [47,48,49,50]. For example, as Marjani et al. clarified, the growth of data provided by IoT has played a remarkable role on the big data horizon [51]. In fact, the adoption of IoT, increases the total volume of the generated data transforming the data into big data [52]. Helping real-time information sharing through autonomous networked actors, IoT data, leads to big data which is characterized by various high-volume, high-velocity, high-variety, and high-veracity features [53]. Regarding these interconnections, the main indicators for “development of positive externalities” for the IoT-Related TIS are a multidisciplinary workforce, similar fields hosting IoT knowledge spillovers, specialized intermediary products, and complementary infrastructures and platforms [37,54]. Accordingly, the final group of hypotheses are proposed as follows:

**Hypothesis** **2g–5g** **(H2g–5g).** *IoT governance functions have a direct and meaningful relationship with the key process of ‘development of positive externalities’ in the IoT-related TIS*.

**Figure 1:**
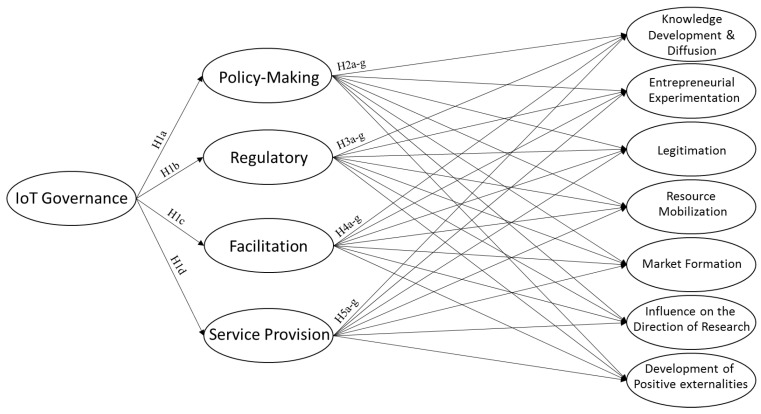
The research’s conceptual model.

Based on the above hypotheses, the conceptual model is proposed in Figure 1.

## 3. Methodology

### 3.1. Research Design

The present study is a descriptive and survey type, and the required information for determining the functions of governance has been gathered using questionnaires, interviews, and an expert panel. Firstly, the library studies and deep interviews were conducted in the fields of governance functions and the IoT-related TIS with academic and industrial experts. Accordingly, the IoT governance and its functions were determined and consequently modified regarding the IoT-related TIS and customized indicators under each of the key processes based on the model provided by Bergek et al. [35]. Conceptualizing the research model in Figure 1, authors created and standardized a questionnaire for gathering and quantifying data. Finally, the collected data were statistically analyzed using SPSS and Smart PLS software. The results were also checked by the experts interviewed at the start point.

### 3.2. Instrument and Data Collection

This research assessed 52 questionnaire items through the research which were used in the previous sections. After modification and adjustment meetings with six professors and experts from government, university, and the industry who had remarkable experience of working in the field of IoT, an experimental survey was piloted. Using Cronbach’s alpha quantity, the reliability of the results was analyzed. The final survey questionnaire consisted of 43 items (Table 2), after eliminating 9 items that did not pass the expected reliability standard.

The authors contacted 440 respondents across the seven industries, including ICT, Healthcare, Transportation, Oil and Gas, Energy, Agriculture, and Banking over three months and received 319 fulfilled questionnaires after excluding 32 incomplete cases (351 in total). All participants had more than one year of experience in dealing with IoT issues either on the technical (24.9%) or non-technical side (75.1%). Their job affiliations were related to government body (40.1%), the industry sector (32.7%), and universities (27.2%). Their roles, in terms of structural components of a TIS, were actors (32.1%), institutions (21.5%), networks (22.8%), and knowledge and products (23.6%). Applying a 5-point Likert scale (from 1 = strongly disagree to 5 = strongly agree), all responses to the questionnaire items were quantified. Finally, statistical explorations were performed on the 319 valid questionnaires and are reflected in the next section.

### 3.3. Structural Equation Modeling (SEM)

SEM is a comprehensive statistical method for investigating the relationships between observed and latent variables [55]. In fact, SEM is a confirmatory method which provides a comprehensive facility for evaluating measurement and structural models. Assessing the validity and reliability of a measurement model are the main functions of the SEM method. In this study, because of non-normal distribution of data, which is explored in SPSS software, the SEM of the relationship between the IoT governance functions and the key processes of the IoT-related TIS has been analyzed using the Partial Least Squares (PLS) method and Smart PLS software.

## 4. Results

### 4.1. Preliminary Tests

After gathering, data were checked for outliers using the Cook’s distance analysis [56]. Results showed that all cases were less than 0.025 and the survey did not encounter outliers. The results of Variance Inflation Factor (VIF) for testing multicollinearity demonstrated that the multivariate assumptions were met [57]. Using t-tests and the Harman’s single factor test, respectively, non-response and Common Method Bias (CMB) also were checked [58,59]. Although the data were gathered over three months, the results of t-tests show that the survey has no non-response bias. The value of 26.8% for the Harman’s single factor, which must be less than 50%, indicates that there is no concern about the CMB and the research results.

### 4.2. Validity and Reliability

Based on the common instructions for testing the validity and reliability of the measurement model, Cronbach’s alpha, factor loadings, composite reliability (CR), and average variance extracted (AVE) were checked [60,61,62]. As shown in Table 3, all the standard values were met.

Hensler et al. proposed a new indicator called the Heterotrait-Monotrait Ratio or HTMT to assess divergent validity [63] instead of using the Fornell-Larker method [64]. HTMT compares the square root value of the AVE with the correlation of two determined constructs. As demonstrated in Table 4, all values were ≤0.9 and the discriminant validity were satisfactory.

### 4.3. Hypothesis Tests

**Figure 2:**
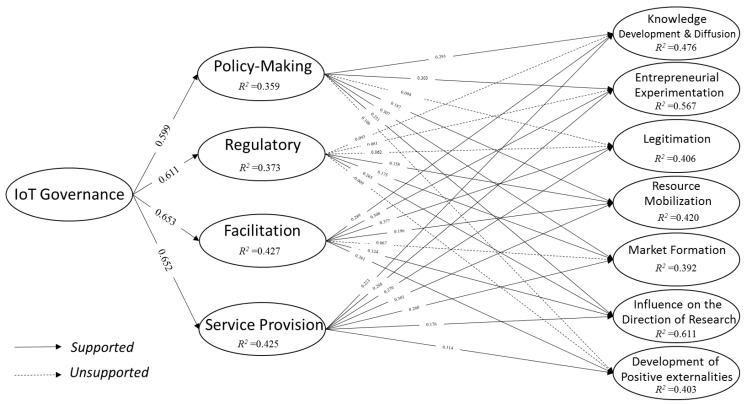
Summary of the research model (see Appendix A (Figure A1 and Figure A2) for more details).

The structural model, including the 32 hypotheses, were tested through the significance coefficients of T between latent variables. If the obtained value was greater than 1.96, and *p*-value < 0.05, that relationship or hypothesis was confirmed. Regarding these standard values, seven paths in Table 5, including H2c (β = 0.094, *p*-value = 0.083), H2g (β = 0.106, *p*-value = 0.053), H3a (β = 0.093, *p*-value = 0.099), H3b (β = 0.081, *p*-value = 0.137), H3c (β = 0.062, *p*-value = 0.333), H3g (β = −0.009, *p*-value = 0.890), and H4e (β = 0.067, *p*-value = 0.235) did not meet the standard value, thus they are not confirmed; all other hypotheses were accepted. The model, including both supported (→) and unsupported (⇢) relationships, is depicted in Figure 2.

The explained variance regarding dependent variables relative to the total variance was measured using the coefficient of determination (R^2^). The results showed that the four IoT governance functions are explained by the IoT governance variable. Furthermore, each of the seven key processes of the IoT-based TIS is well-explained by the functions of IoT governance. Finally, according to Equation (1) proposed by Tenenhaus et al. [65], the Goodness Of Fit (GOF) for total model has been measured based on the geometric mean of the average communality and the average R^2^ (Table 6). The 0.442 value for GOF, which is >0.36, shows that the study model has a strong fitness.

(1) $$\mathrm{GOF}=\sqrt{\bar{\mathrm{Communality}}\times \bar{R^{2}}}$$

## 5. Discussion

This study has investigated the relationship between IoT governance and key processes of the IoT innovation system in Iran. Regarding this, four functions for IoT governance, and seven key processes of the IoT-related TIS were considered. A conceptual model, including 12 variables and 32 hypotheses was structured and measured to examine the direct relationships, of which 25 cases were supported through SEM. The results could be the basis of future research and policy solutions in developing countries.

According to the analytical results, the IoT governance affects the IoT innovation system through all the functions presented by previous macro-level studies for governance concepts. Thus, confirming the research conducted by Hillman, governance occurs outside of the key processes of a TIS [15]. It seems that governance uses the function of policy-making for the strategic issues and applies the other three functions in administrative areas. In fact, executive functions are used to achieve comprehensive goals and programs set at the strategic level by IoT policymakers. Consistent with previous studies [66,67], authors believe that executive functions of IoT governance can be delegated to non-governmental sectors and be conducted collaboratively. These issues were the introduction to discovering the direct relationship between governance functions and key processes of the IoT innovation systems which are the most important achievement and research contributions. Most scientific studies on TIS, which originate from developed countries and focuses primarily on functional analysis for non-digital industries, have often approached the governance issue as an integrated part of its functions instead of interpreting it as a concept that affects TIS from the outside [15]. Furthermore, in recent systemic approaches to innovation, including National, Sectoral, Regional, and Technological [11,12,13,14], there is no evidence of considering the role of governance as an outside variable. Authors believe that the provided model has well positioned the governance outside a TIS, and the supported hypotheses can be considered in the literature of TISs in the era of Industry 4.0.

Based on the results, the governance’s policy-making function directly affects most of the key processes in the IoT-related TIS. Authors believe that in the process of “knowledge development and diffusion”, if some feasibility studies are carried out across technical layers such as sensors, networks, and platforms, it brings appropriate, safe, and reliable solutions for the society and community of practices. Consequently, privacy concerns, security, and other IoT challenges will be desirably addressed. In addition, definable goals and regulations under the key process of “influence on the direction of research” require policymakers’ attention to ensure that policies are correctly designed. Prioritizing IoT applications for the “entrepreneurial experimentations”, formulating and communicating public procurement programs for “market formation”, and the policy of training a specialized workforce through educational and academic programs under “resource mobilization” are other issues that should be nationally addressed by policymakers. All of these align with policy-making sub-functions developed by Abert [31].

Contrary to policy-making, the regulatory function directly affects fewer key processes of the IoT innovation system. Applying the technical and business standards for what is policy made and implemented on the one hand, and what is wanted by the market parties on the other hand, the regulator, which can be the government or its affiliated institution, plays a critical role in the national “market formation” of IoT. For example, dedicating subsidies to the supply and demand side requires regulatory interventions in the market to secure their interests, which must be considered by the governance. The results, that overlap with Levi-Faur’s findings [32], also showed that the IoT governance affected the process of “influence on the direction of research” through its regulatory actions to guide development activities and correct the implementation of policies. Finally, the role-playing of regulation in the “resource mobilization” processes is in line with economic and social interventions of the regulatory function of the IoT governance [29].

According to the modeling results, all key processes in the IoT innovation system, except the “market formation”, are directly affected by the facilitation function of governance. This means that the existence of an appropriate platform and infrastructure has a remarkable role in developing the IoT innovation system [25] and should be considered by the IoT governance. The authors’ point of view, similar to the new governance concept developed by Rhodes [68], is that the actual governance here is that the development of both the technical and business infrastructures should be conducted in collaboration with the structural components of the IoT-related TIS. In the absence of internal capability in the development of some cases, particularly for a developing country, the service provision function of IoT governance will be enabled.

The IoT governance through the provision of products and services is the only function that directly affects all seven key processes within the IoT innovation system. Authors, by inspiration of previous research conducted by Batly et al. [69], believe that the purpose of this function is to ensure the wellbeing of all stakeholders in the IoT technological innovation system and even to support some commonalities with similar areas. For example, the process of “development of positive externalities” into other digital technologies such as big data, artificial intelligence, cloud computing, etc., needs the provision of infrastructure even from abroad, especially if the facilitation function of IoT governance has no convincing response.

## 6. Conclusions and Recommendation

Developing countries need the IoT governance in order to develop safe and secured technological innovations following the Industry 4.0. A framework that can intelligently affect the key processes of developing a technological innovation in cooperation with the system’s structural components. This study has investigated the relationship between the functions of IoT governance and the key processes of the IoT in Iran. According to the research findings, IoT governance influences seven key TIS processes through four common functions. Emphasizing the normative and ideological aspects of the working, industrial, and human society, the function of policy-making can mainly affect the two processes of “development and diffusion of knowledge” and “influence on the direction of research”. The function of regulation, relying on the economic, technological, and social interventions, as well as using standard tools, plays a key role in the process of “market formation” to ensure the implementation of adopted policies. Finally, IoT governance can affect almost all key processes in the IoT innovation system through facilitation and service provision. Exploring and modeling these determining direct relationships between the IoT governance functions and the key processes of the IoT innovation system in Iran, as a developing country, is the most important achievement and research contribution of this study. In fact, the provided model has well positioned the governance outside a TIS, and the supported hypotheses can be considered in the literature of TISs in the era of Industry 4.0 by following researchers.

Future studies can test the results of this study at other levels of innovation systems such as regional, national, or sectoral. In addition, applying or evaluating the governance concept for similar fields, including artificial intelligence, big data, blockchain, and other digital technologies could be suggested as a research topic for those who are interested in this field. It is necessary to mention at this point that this study has focused solely on the direct relationships between the IoT governance, the IoT governance functions, and the key processes of the IoT innovation system. Hence, some governance functions may have indirect relationships with the key processes of the IoT-based TIS and should not be neglected by the IoT governance. Thus, considering indirect relationships between the IoT governance functions and key processes of the IoT-related TIS could be suggested as a research topic for following researchers.

In terms of practical implications, this study proposes that adopting and implementing the IoT technology in a developing country needs full coordination and cooperation at the first steps of development at the national level. The IoT actors, networks, players often coming from government sector in developing countries, and institutions should actively participate in shared activities. Although these activities, in this study, have been grouped in seven key processes under the IoT–related TIS, it is very important to pay attention to prioritize critical processes. In fact, decision makers in developing countries, after analyzing functional dynamics in their own context, should decide the governance arrangements regarding the seven key processes of the IoT-related TIS at the national level. However, it seems that the key process of “legitimation”, because of the unbalanced economy, is playing a vital role in the IoT Iran innovation system. In fact, the pervasive acceptance of IoT in Iran could enable other processes in short term, including “Knowledge Development & Diffusion”, “Entrepreneurial Experimentations”, and “Direction of Research”. Relying on these four processes in practice, the other three processes could emerge in the long term.

The main limitation of this study was the collection of data through questionnaire, as some respondents may have provided inaccurate responses.

## Figures and Tables

**Table 1:** TIS processes and IoT criteria.

Bergek et al. [35]	Hakkert et al. [36]	Description [15]	IoT-Based Criteria [37] (* by Authors)
Knowledge development and diffusion	Knowledge development	Creation of breadth and depth of knowledge base of TIS	Training of professionals Conducting promotion campaigns Organizing conference/workshops/seminars Demonstrations & exhibitions
	Knowledge diffusion through networks	Diffusion and combination of knowledge	Conducting feasibility studies IoT market research & assessment Developing complementary technologies Network of technology & research cooperation
Entrepreneurial experimentation	Entrepreneurial activities	Existence of incentives and/or pressures for actors to enter TIS and to direct their activities towards certain parts of TIS	Experimentation of new applications of IoT Launching pilot IoT projects Entry of firms to IoT markets System for innovation & incubation
Legitimation	Creation of legitimacy/counteract resistance to change	Legitimacy is a matter of social acceptance and compliance with relevant institutions	Strength of lobby actions Rise & growth of interest groups Social acceptability IoT related institutions *
Resource mobilization	Resources mobilization	Extent to which TIS is able to mobilize competence/human capital and financial capital	Providing R&D budgets, grants & loans Launching IoT related education programs Mobilizing human resources Funding scale up on IoT projects
Market formation	Market formation	Articulation of demand, the existence of standards and timing, size, and type of markets actually formed	Providing subsidies Government procurement programs Regulatory reform Standardizations
Influence on the direction of search	Guidance of the search	Probing into new technologies and applications in an entrepreneurial manner	Setting collective goals for IoT development Design of favorable rules and regulations Publicizing expectations Providing direction of development
Development of Positive externalities/external economies	-	Generation of positive external economies	Multidisciplinary workforce * Similar fields hosting IoT knowledge spillovers * Specialized intermediary products * Complementary infrastructures & platforms *

**Table 2:** Questionnaire items.

Constructs	Items
IoT Governance	IG1: IoT governance plays a crucial role in the development of this technology in the country IG2: IoT issues of privacy, security, and public security need governance IG3: Paying attention to the interests of various IoT stakeholders is a governance’s duty
Policy Making	PM1: The national development of IoT requires policy-making PM2: Activities and actions related to the IoT development should be nationally prioritized PM3: Social values should be considered by IoT decision and policy makers
Regulatory	RG1: Implementing IoT at the national level requires the intervention of the government or its representative(s) RG2: The national development of IoT requires the establishment of laws, regulations, & specific standards RG3: Strengthening the IoT service providers requires the specific regulation
Facilitation	FC1: The national development of IoT platform and infrastructure needs governance FC2: Empowering IoT services and products providers is a governance’s duty FC3: The allocation of resources for the national IoT development needs governance
Service Provision	SP1: Supplying the IoT required products and services should be conducted by governance SP2: Collaboratively procurement programs of supplying IoT infrastructure need governance SP3: Required products and services for the national development of IoT can be supplied from abroad
Knowledge Development & Diffusion	KD1: Creating and spreading the IoT related knowledge in the country is essential KD2: The IoT market assessment and research plays a key role in the national IoT development KD3: IoT exhibitions, conferences, seminars, and workshops helps to spread the IoT-related knowledge KD4: Research and technology cooperation accelerate the development of the IoT technology
Entrepreneurial Experimentations	EX1: IoT-based products, services, and applications require special tests before entering the market EX2: Pilot projects significantly contribute to the success of IoT development in the country EX3: The entry of IoT-related service providers into the market will lead to the IoT development in the country EX4: Having an innovation system and dedicated incubators is essential for the IoT entrepreneurial experiences
Legitimation	LM1: Social acceptance of the IoT products and services accelerates the development of IoT innovation system LM2: The existence of dedicated institutions is essential for developing IoT in the country LM3: The emergence and growth of groups interested in IoT development contributes to the IoT legitimacy LM4: The power of lobbying among the IoT innovation system’s actors creates legitimacy for IoT
Resource Mobilization	RM1: Allocation and equipment of needed resources are essential for IoT development in the country. RM2: Financing IoT projects in the post-pilot phase helps to develop IoT at the national level RM3: Designing the educational programs to train the IoT experts should be considered across the country RM4: Financing through R&D budgets, grants, and loans helps to develop IoT nationally
Market Formation	MF1: Intelligent link between supply & demand plays a vital role in shaping the IoT market in the country MF2: Providing subsidies focusing on the national development of IoT products & services is essential MF3: Customizing current regulations or making new regulations is critical for developing IoT in the country MF4: National procurement programs should be aimed at strengthening IoT service providers
Influence on the Direction of Research	DR1: The national development of IoT requires a comprehensive program approved by actors & stakeholders DR2: Designing favorable regulations will help the IoT development in the country DR3: Publicizing of expectations of actors and stakeholders leads to a good direction of IoT researches DR4: Developing IoT-based products, services, and technologies requires monitoring and guidance
Development of Positive externalities/External economies	DE1: IoT spillovers into other fields indicate the proper development of an innovation system for IoT DE2: The national development of IoT leads to the combined and multi-skilled workforce DE3: There are significant similar areas for the IoT knowledge spillovers DE4: IoT platforms and infrastructures have substantial commonalities with other digital technologies

**Table 3:** Internal and Convergent reliability.

Construct	Items	Factor Loading ≥ 0.4	Cronbach’s alpha ≥ 0.7	CR ≥ 0.7	AVE ≥ 0.5
IoT Governance	IG1	0.873	0.814	0.890	0.730
	IG2	0.882			
	IG3	0.805			
Policy Making	PM1	0.849	0.796	0.880	0.711
	PM2	0.864			
	PM3	0.815			
Regulatory	RG1	0.856	0.758	0.861	0.675
	RG2	0.825			
	RG3	0.781			
Facilitation	FC1	0.776	0.739	0.852	0.657
	FC2	0.833			
	FC3	0.822			
Service Provision	SP1	0.867	0.747	0.858	0.672
	SP2	0.679			
	SP3	0.896			
Knowledge Development & Diffusion	KD1	0.701	0.774	0.856	0.600
	KD2	0.844			
	KD3	0.840			
	KD4	0.702			
Entrepreneurial Experimentations	EX1	0.840	0.876	0.915	0.729
	EX2	0.828			
	EX3	0.886			
	EX4	0.859			
Legitimation	LM1	0.811	0.827	0.889	0.672
	LM2	0.922			
	LM3	0.896			
	LM4	0.613			
Resource Mobilization	RM1	0.853	0.881	0.918	0.737
	RM2	0.867			
	RM3	0.859			
	RM4	0.856			
Market Formation	MF1	0.783	0.807	0.874	0.634
	MF2	0.801			
	MF3	0.754			
	MF4	0.846			
Influence on the Direction of Research	DR1	0.790	0.748	0.842	0.573
	DR2	0.819			
	DR3	0.656			
	DR4	0.752			
Development of Positive externalities	DE1	0.807	0.796	0.868	0.623
	DE2	0.787			
	DE3	0.850			
	DE4	0.706			

**Table 4:** Discriminant validity.

Construct	IG	PM	RG	FC	SP	KD	EX	LM	RM	MF	DR	DE
IG												
PM	0.744											
RG	0.776	0.401										
FC	0.839	0.594	0.698									
SP	0.837	0.478	0.809	0.523								
KD	0.784	0.684	0.621	0.738	0.660							
EX	0.762	0.690	0.635	0.749	0.704	0.628						
LM	0.638	0.477	0.581	0.714	0.624	0.445	0.645					
RM	0.621	0.516	0.616	0.591	0.669	0.577	0.616	0.475				
MF	0.651	0.611	0.598	0.520	0.652	0.656	0.562	0.318	0.610			
DR	0.827	0.706	0.824	0.868	0.746	0.707	0.753	0.519	0.627	0.703		
DE	0.628	0.495	0.536	0.713	0.648	0.720	0.518	0.403	0.482	0.586	0.722	

**Table 5:** Summary of the results.

Hypotheses	Path	(*β*)	T-Statistics	*p*-Value	Result
H1a	IG → PM	0.599	16.067	0.00	Supported
H1b	IG → RG	0.611	13.234	0.00	Supported
H1c	IG → FC	0.653	16.140	0.00	Supported
H1d	IG → SD	0.652	17.589	0.00	Supported
H2a	PM → KD	0.293	5.980	0.00	Supported
H2b	PM → EX	0.303	6.232	0.00	Supported
H2c	PM → LM	0.094	1.738	0.083	Unsupported
H2d	PM → RM	0.187	3.424	0.001	Supported
H2e	PM → MF	0.307	6.156	0.00	Supported
H2f	PM → DR	0.251	5.826	0.00	Supported
H2g	PM → DE	0.106	1.942	0.53	Unsupported
H3a	RG → KD	0.093	1.651	0.099	Unsupported
H3b	RG → EX	0.081	1.488	0.137	Unsupported
H3c	RG → LM	0.062	0.968	0.333	Unsupported
H3d	RG → RM	0.158	2.348	0.019	Supported
H3e	RG → MF	0.175	3.006	0.003	Supported
H3f	RG → DR	0.265	5.016	0.00	Supported
H3g	RG → DE	−0.009	0.139	0.890	Unsupported
H4a	FC → KD	0.289	6.163	0.00	Supported
H4b	FC → EX	0.308	7.718	0.00	Supported
H4c	FC → LM	0.277	6.840	0.00	Supported
H4d	FC → RM	0.196	4.082	0.00	Supported
H4e	FC → MF	0.067	1.190	0.235	Unsupported
H4f	FC → DR	0.324	8.115	0.00	Supported
H4g	FC → DE	0.381	6.252	0.00	Supported
H5a	SP → KD	0.223	4.108	0.00	Supported
H5b	SP → EX	0.288	5.992	0.00	Supported
H5c	SP → LM	0.270	4.590	0.00	Supported
H5d	SP → RM	0.301	5.201	0.00	Supported
H5e	SP → MF	0.260	4.365	0.00	Supported
H5f	SP → DR	0.176	3.714	0.00	Supported
H5g	SP → DE	0.314	4.853	0.00	Supported

**Table 6:** Total fitness with GOF.

Variables	R^2^	Communality
IoT Governance	-	0.444
Policy Making	0.359	0.410
Regulatory	0.373	0.347
Facilitation	0.427	0.315
Service Provision	0.425	0.356
Knowledge Development & Diffusion	0.476	0.340
Entrepreneurial Experimentations	0.567	0.538
Legitimation	0.406	0.340
Resource Mobilization	0.420	0.548
Market Formation	0.392	0.389
Direction of Research	0.611	0.291
Development of Positive Externalities	0.403	0.372
Average	0.442	0.401
$\mathrm{GOF}=\sqrt{\bar{\mathrm{Communality}}\times \bar{R^{2}}}=\sqrt{0.401\times 0.442}=0.421$		

**Figure A1:**
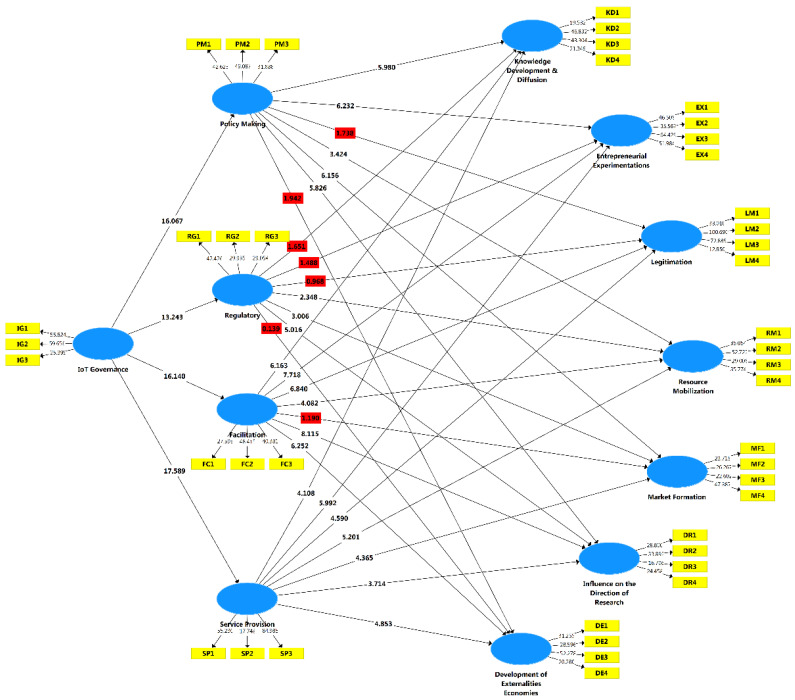
The measurement model.

**Figure A2:**
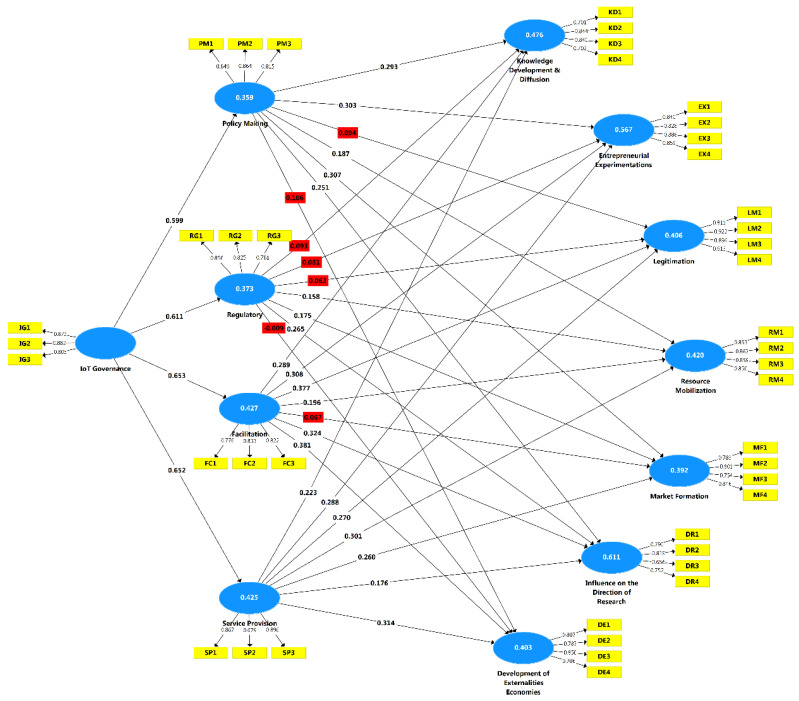
The structural model.

## Data Availability

Not applicable.
